# Supplementary material for: Electron heating and thermal relaxation of gold nanorods revealed by two-dimensional electronic spectroscopy
Source: Nat Commun. 2018 Mar 1;9:891. doi: 10.1038/s41467-018-03002-8 (PMC5832861; doi:10.1038/s41467-018-03002-8)
Supplement: Supplementary file 1 — Supplementary Information [file 41467_2018_3002_MOESM1_ESM.pdf]

Supplementary Information for

Electron heating and thermal relaxation of gold nanorods revealed by two-dimensional electronic spectroscopy

Aude Lietard[1,2], Cho-Shuen Hsieh[1,2], Hanju Rhee[3], and Minhaeng Cho[1,2]

[1] *Center for Molecular Spectroscopy and Dynamics, Institute for Basic Science (IBS), Seoul, 02841, Republic of Korea*

[2] *Department of Chemistry, Korea University, Seoul, 02841, Republic of Korea*

[3] *Seoul Center, Korea Basic Science Institute (KBSI), Seoul, 02841, Republic of Korea*

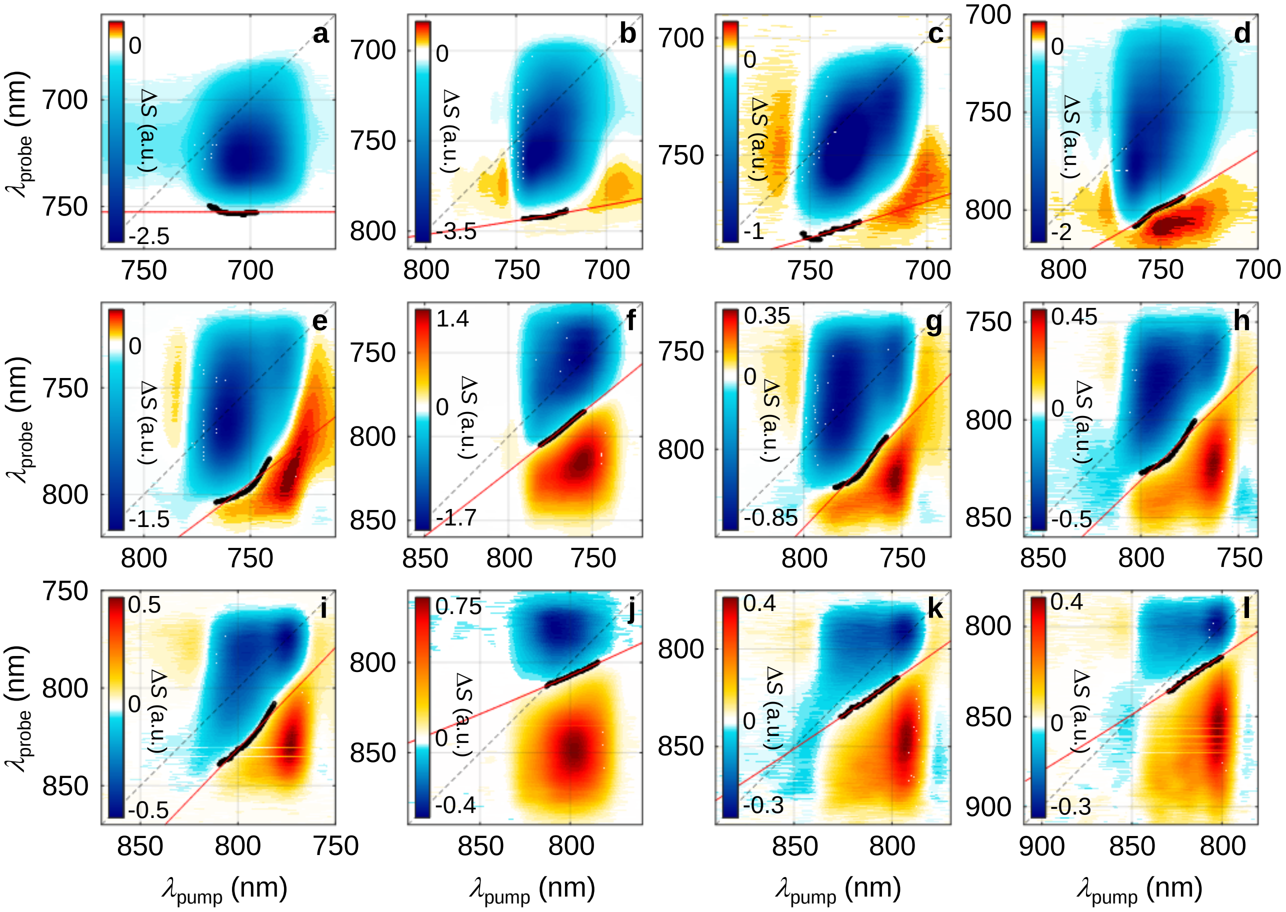

Supplementary Figure 1: AuNR LgSPR 2D electronic spectra at waiting time of 1 ps. The 2D electronic spectra of the AuNRs measured at T_w=1 ps. The center wavelengths of both the pump and probe pulses are (**a**) 700 nm; (**b**) 720 nm; (**c**) 730 nm; (**d**) 740 nm; (**e**) 750 nm; (**f**) 760 nm; (**g**) 770 nm; (**h**) 780 nm; (**i**) 790 nm; (**j**) 800 nm; (**k**) 810 nm and (**l**) 820 nm. The thick black line in each panel is a collection of nodal points at which the 2D signal is zero. The red line is the linear fit to the nodal points. The dependence of the nodal line slope on pump center wavelength is shown in Fig. 4**g** in the main text.

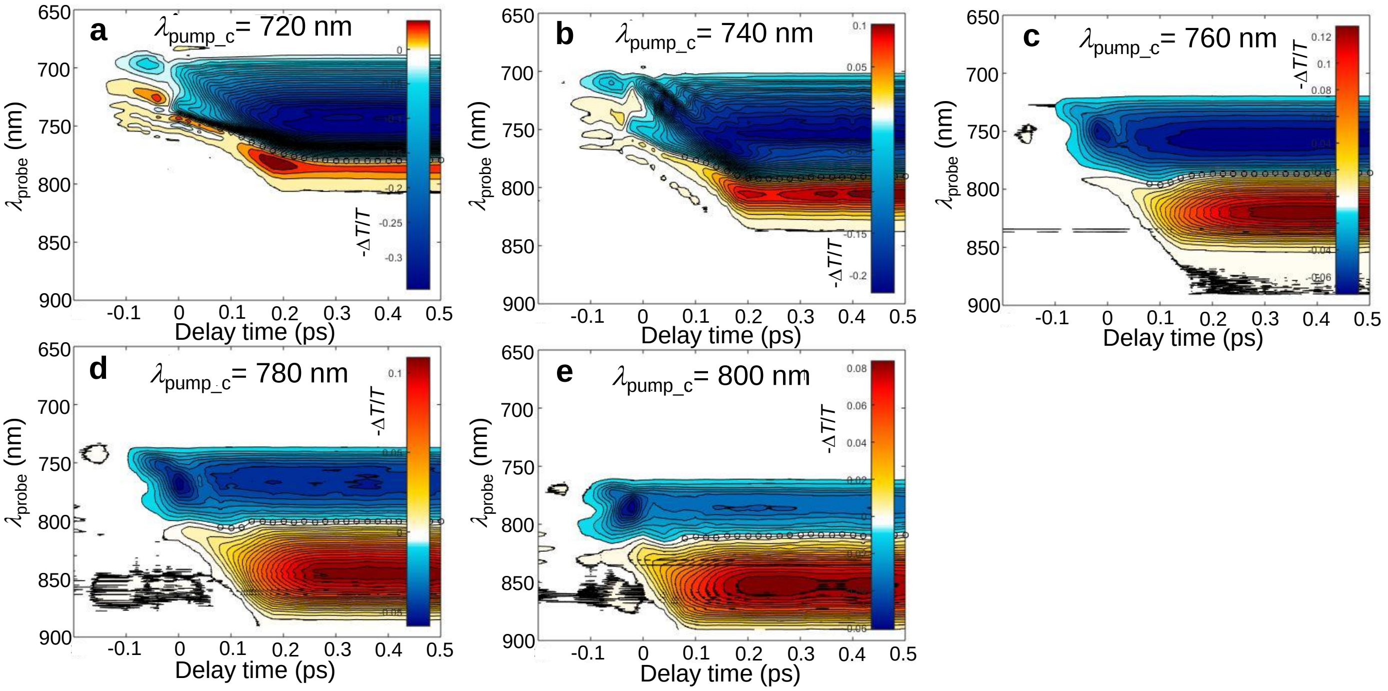

Supplementary Figure 2: Time-dependent transient absorption spectra: Short time domain. The time and frequency-resolved TA spectra at short times (< 0.5 ps). The center wavelength (λ_{pump_c}) of the pump pulse varies from 720 to 800 nm (**a**-**e**). The color bar represents the differential transmitted intensity ($-\Delta T$) normalized by the probe intensity (T) without the pump, that is, $-\Delta T/T$=$-(I_{pump\text{-}on}- I_{pump\text{-}off})/I_{pump\text{-}off}$, where $I_{pump\text{-}on}$ and $I_{pump\text{-}off}$ are the transmitted probe intensities with and without the pump beam, respectively.

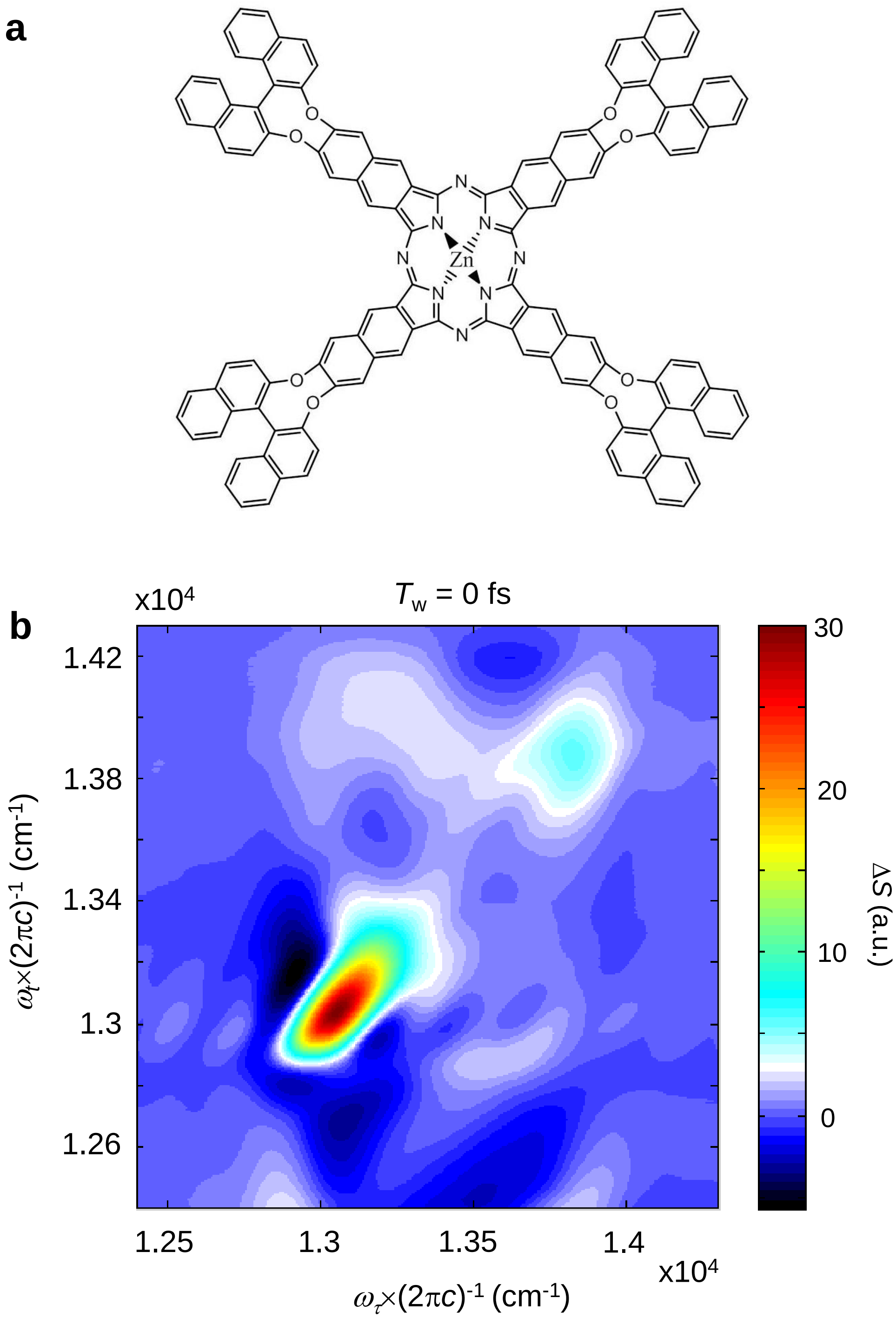

Supplementary Figure 3. 2D electronic spectrum of zinc naphthalocyanine derivative at zero waiting time. (**a**) The chemical structure of zinc-naphthalocyanine derivative with four binaphtholate groups. (**b**) The two-dimensional electronic spectrum of the zinc naphthalocyanine derivative (**a**) dissolved in tetrahydrofuran measured at T_w = 0 fs.[1] Unlike the 2DES spectra of the AuNRs at negative and short waiting times, it is clear that the 2DES spectrum of this organic dye molecule does not show any notable (diagonally elongated) fringe or spectral interference pattern at all. This indicates that the coherent electronic nonlinear susceptibility is not sufficiently large enough to diffract the TG signal.

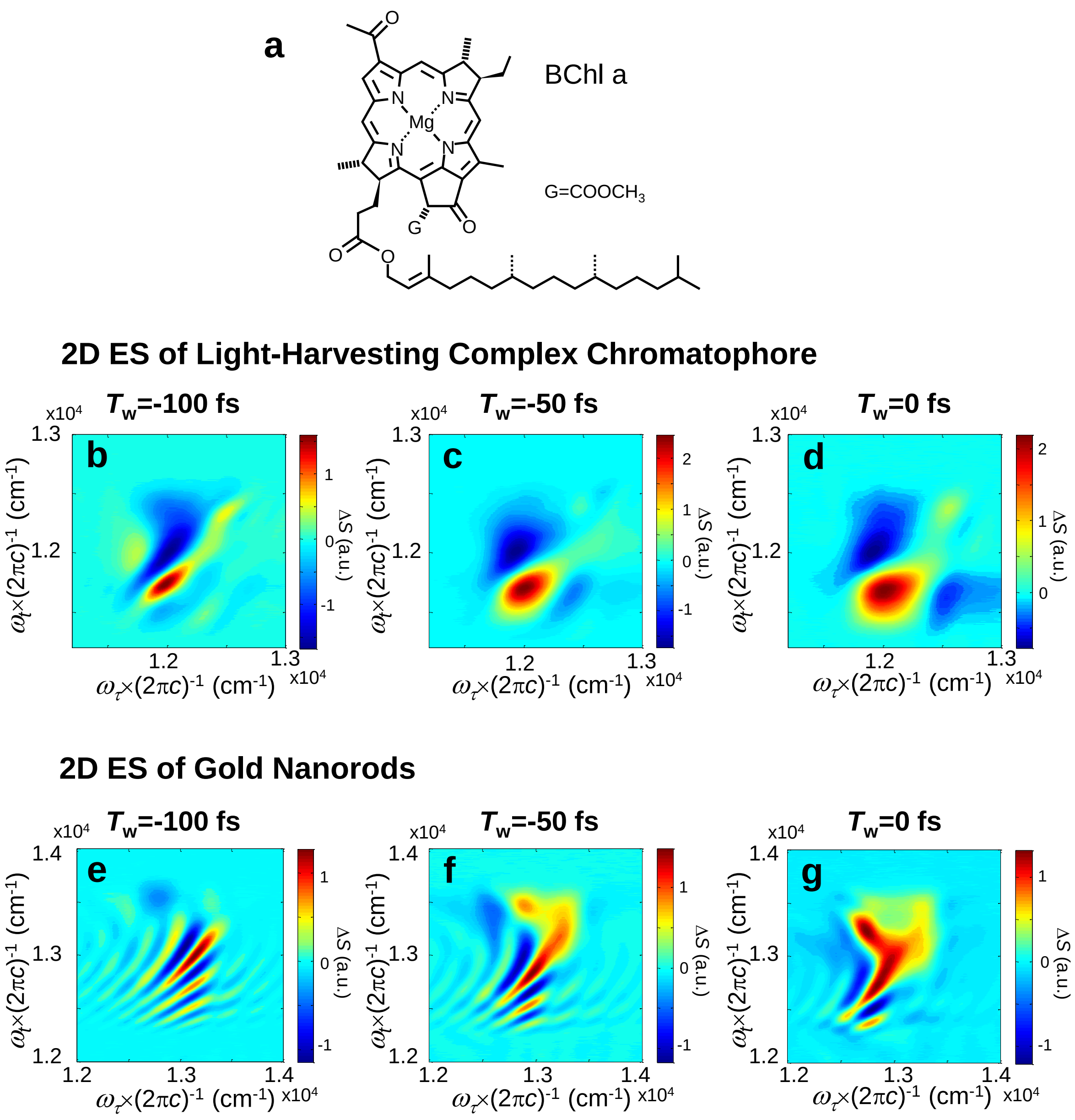

Supplementary Figure 4: 2D electronic spectra of light-harvesting complex (LHC) chromatophore isolated from *Rhodobactor sphaeroides* bacteria and AuNR measured at negative waiting times. (**a**) The chemical structure of bacteriochlorophyll a (BChl a) that is the main constituent chromophore in light-harvesting complex (LHC). Comparison between the 2DES spectra of (**b-d**) LHC chromatophore isolated from *Rhodobactor sphaeroides* bacteria and (**e-g**) AuNR measured at different waiting times, T_w = -100 fs (**b**,**e**), -50 fs (**c**,**f**) and 0 fs (**d**,**g**). In stark contrast with the spectral interferences observed in the 2DES spectra of AuNRs at negative and short waiting times, the 2DES of the LHC (bio-organic system) do not show strong and dense fringe patterns at all. Here, it should be emphasized that the pump and probe polarizations were deliberately controlled to be perpendicular to each other in the present 2DES measurement on AuNRs and furthermore one more polarizer whose transmission axis is parallel to the probe polarization is placed in front of the detector for the 2DES measurement of AuNR to remove any contamination of 2DES signal due to scattering of pump pulses by AuNRs or diffracted TG signal. With this specially designed perpendicular polarization geometry, a large pump scattering signal could be effectively removed. Nevertheless, a considerable fringe signal created by interference between the probe and delayed TG signal generated by the TG is observed in the 2DES of AuNR, which clearly indicates that AuNR has an exceptionally strong nonlinear SPR response in contrast to organic molecules (both naphthalocyanine dye and chlorophyll in LHC) that usually have tightly bound electrons to nuclei. Note again that the free electron-like behaviors of SPR excited electrons during the probe pulse duration time are the key for the exceptionally large but transient scattering cross section of AuNRs. This has never been observed before, because the transient absorption, a pump-probe measurement method, cannot reveal the spectral interference patterns observed in our 2DES spectra, because the pump-probe spectrum is nothing but a projected average one-dimensional spectrum of the 2DES spectrum onto the *y*-axis (ω_t-axis) in the 2DES spectrum.

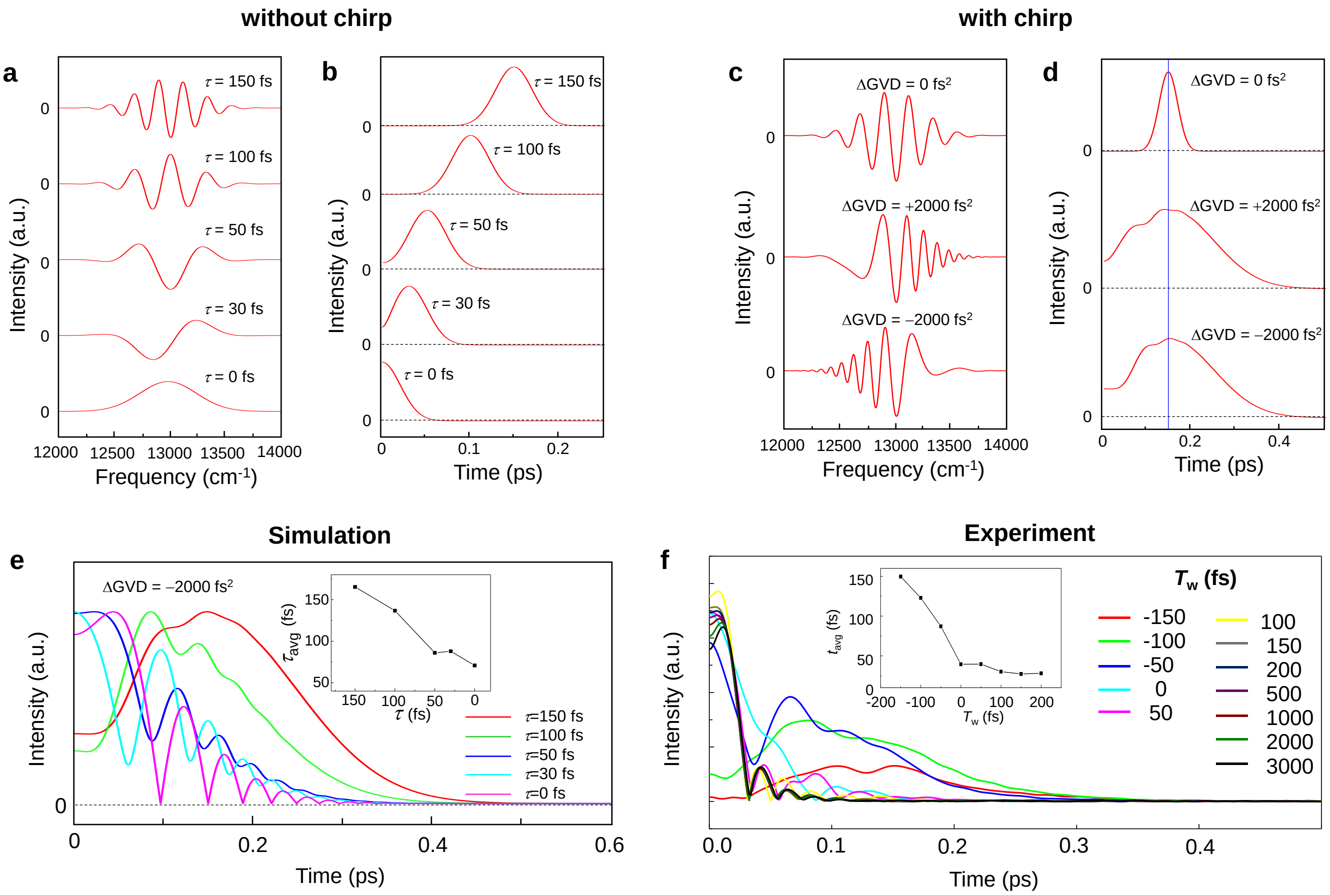

Supplementary Figure 5: Simulated spectral interferograms and Fourier transformed signals in time domain. Simulated spectral interferograms (**a** and **c**) created by the interference of time-separated two Gaussian pulsed electric fields (E_1 and E_2) with time delay of τ and their Fourier transformed signals (**b** and **d**) in the time domain. The two electric fields are given as $E_{1,2}(\omega)=E_0\times\exp[-2\ln(2)\times(\omega-\omega_0)^2\times(\Delta\omega_{FWHM})^{-2}]\times\exp\{i\Phi_{1,2}(\omega)\}$, where E_0 is the electric field amplitude, ω_0 (12987 cm^{-1}) the center frequency of their spectra, $\Delta\omega_{FWHM}$ (600 cm^{-1}) the full width at half maximum of their power spectra, and $\Phi_{1,2}(\omega)$ the frequency dependent phases. For a transform-limited pulse without chirp, $\Phi(\omega)=0$. On the other hand, when E_1 and E_2 have finite group delay dispersions (GDD$_1$ and GDD$_2$, respectively), $\Phi(\omega)$ is then given as $\Phi_{1,2}(\omega)=$ GDD$_{1,2}\times(\omega-\omega_0)^2/2$. (**a**) Spectral interferograms of time-separated transform-limited electric fields $E(\omega)=E_1(\omega)\times\exp(i\omega\tau)+E_2(\omega)$ as varying τ from τ=150 fs (top) to τ=0 fs (bottom) and (**b**) the corresponding Fourier-transformed signals in time domain.[2-6] It should be noted that the heterodyne term, $2\mathrm{Re}[E_1(\omega)E_2(\omega)^*\times\exp(i\omega\tau)]$, is only considered here in the spectral interferogram, because the other homodyne terms are constant without producing any interference pattern. As τ increases, the fringe of the spectral interferogram becomes dense as can be seen in (**a**), while the temporal position of the Fourier-transformed signal pulse in (**b**) which exactly matches the time delay (τ) between E_1 and E_2, shifts away from zero as expected. (**c**) Spectral interferograms of time-separated two electric fields with different net GDD (ΔGDD= GDD$_1$-GDD$_2$) at τ=150 fs and (**d**) the corresponding Fourier-transformed signals. Top: ΔGDD = 0 fs^2 (transform-limited), Middle: ΔGDD = +2000 fs^2 (positive chirp), Bottom: ΔGDD = –2000 fs^2 (negative chirp). Note that, in the case that ΔGDD>0, the fringe becomes denser as the frequency increases, while the frequency-dependent fringe pattern is inversed for ΔGDD<0. The corresponding FT in time-domain shown in (**d**) is broad in time when the pulsed field has a chirp. (**e**) Fourier-transformed signals obtained from the spectral interferograms of time-separated two electric fields with ΔGDD = –2000 fs^2 as varying τ from τ=150 fs to τ=0 fs. Inset: the average (first moment) of time (τ_{avg}) calculated from the Fourier-transformed positive-part signals of chirped pulses (ΔGDD = –2000 fs^2) vs the set time delay (τ). Note that τ_{avg} is almost linearly proportional to τ. (**f**) From the experimentally measured fringes extracted from the 2DES spectra, the Fourier-transformed signals in time domain are plotted here. Then, the first moment (average) of time τ_{avg} from the time-domain signal is compared with the waiting time. This comparative investigation shows that the pulses are chirped and the TG signal induced by the LgSPR optical nonlinearity interferes with the probe pulse at the detector.

Supplementary Table 1. The characteristic time constants of the photo-induced processes of AuNRs obtained from the time evolution of the positive TA peak as a function of the center pump wavelength (λ_{pump_c}). The values in parentheses are the corresponding time constants obtained from the time evolution of the negative TA peaks (Figs. 1**b-f**). $t_{e\text{-}ph}$ and $t_{ph\text{-}ph}$: e-ph and ph-ph relaxation times, respectively. T_{osc}: period of the slowest oscillation component. t_{damp}: damping time of the oscillation.

λ_{pump_c}	720 nm	740 nm	760 nm	780 nm	800 nm
$t_{e\text{-}ph}$ (ps)	(4)	5 (4.9)	2.4 (2.8)	2.6 (3.2)	3.5 (3.6)
$t_{ph\text{-}ph}$ (ps)	59 (59)	53 (72)	58 (93)	75 (122)	57 (114)
T_{osc} (ps)	57	57	54	55	57
t_{damp} (ps)	-	58	44	51	45

Supplementary Note 1: Transient grating and 2DES interference spectrum at negative waiting time

The two-dimensional electronic spectroscopy (2DES) is a four-wave-mixing process and the first three radiation-matter interactions are electric dipole-electric field interactions for molecular systems. Experimentally, one can measure the 2DES signal electric field amplitude and phase with respect to delay times between involved laser pulses. In the present pump-probe-type 2DES, the generated signal field interferes with probe and the interference term is only detected. This is often called the self-heterodyne-detection technique.

Normal 2DES measurement (T_w > 0). The pump-probe-type 2DES experimental setup used in the present work is shown in Fig. 2**a**. A pair of twin pump pulses is generated by using a pulse shaper (Dazzler), which are denoted as pu1 and pu2 in Supplementary Figure 6. The time delay between pu1 and pu2 is defined as τ. Then, the probe pulse of which propagation direction is determined by its wavevector $\mathbf{k}_{pr}$ is delayed from the pu2 by a waiting time T_w. After three field-matter interactions with nonlinear optical material (AuNRs in the present work) with three pulses, the third-order polarization created in the material generates the 2DES signal field (see Supplementary Figure 6), which interferes with probe because they propagate co-linearly and are detected by the spectrograph. The interference term between the 2DES signal field and the probe is detected by using an array detector.

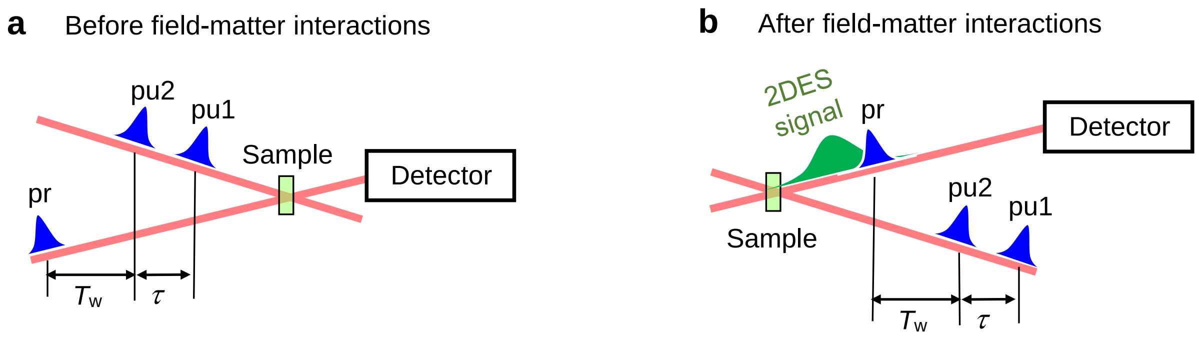

Supplementary Figure 6: Pulse sequence for 2DES at positive waiting time. The incident laser beam and generated 2DES signal pulse sequences (**a**) before and (**b**) after field-matter interactions at a positive waiting time (T_w > 0).

Since the general theory on the 2DES was presented before and can be found in literatures,[7] we shall not provide any further theoretical description on this normal 2DES in a pump-probe-type geometry. Our experimentally measured 2DES spectra (Fig. 4) at positive waiting times show both positive and negative peaks with nodal line separating the two peaks. Detailed analysis results are provided in the main text.

2DES spectral features in negative waiting time domain (T_w < 0). Although the 2DES spectra at positive waiting times exhibit normal spectral patterns, our 2DES spectra taken at negative waiting times show complicated but non-random 2D spectral interference (fringe) patterns. One cannot quantitatively measure such spectral interference (fringe) patterns in one-dimensional (in frequency) pump-probe spectra because the latter corresponds to the probe-frequency-dependent spectrum that can be obtained by projecting the 2DES onto the y-axis (probe frequency axis). Such oscillatory interference patterns in a given 2DES spectra at negative waiting times completely disappear in the corresponding pump-probe spectrum even at the same negative waiting time, because the oscillating components are averaged out.

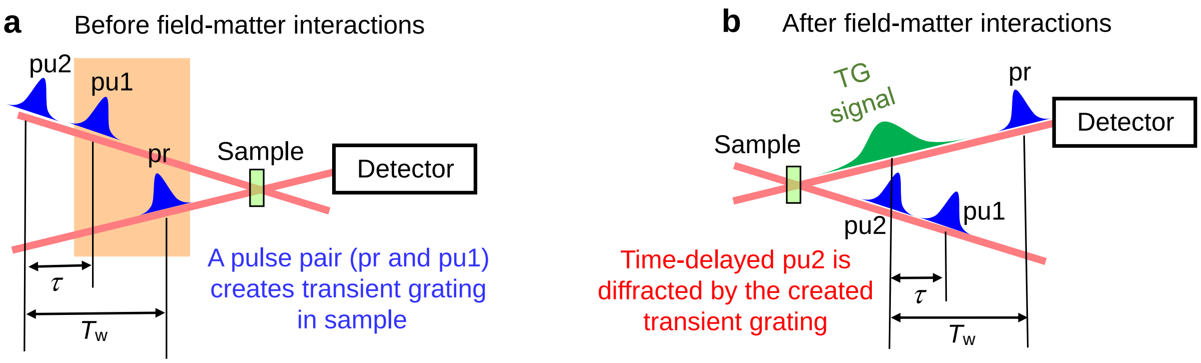

Supplementary Figure 7: Pulse sequence for 2DES at negative waiting time. The incident laser beam and generated TG signal pulse sequences (**a**) before and (**b**) after field-matter interactions at a negative waiting time ($T_w < 0$).

To understand the highly oscillating interference patterns in our 2DES spectra at negative waiting times, one should consider the pulse sequence and 4WM field-matter interactions when the waiting time is negative. In Supplementary Figure 7, the pulse sequences before and after field-matter interactions are schematically drawn. From the definition of the waiting time, which is the time difference of the probe pulse from the second pump pulse (pu2), the pulse sequence shown in Supplementary Figure 7 corresponds to the case that the probe pulse precedes the second pump pulse (pu2). Therefore, the pulse configuration in Supplementary Figure 7 is the case when the waiting time is negative. When 2DES signal (and spectrum) is measured, the delay time τ is scanned from 0 to a finite time. When the pair of pulses, probe and pump 1 (pu1), that propagate differently in space, interact with nonlinear optical sample, a transient grating (TG) is generated inside the sample. Then, the second pump pulse (pu2) can be diffracted by thus created transient grating and the diffracted third-order TG signal field of which amplitude is proportional to the amplitudes of probe, pump 1 (pu1), and pump 2 (pu2) is produced (see Supplementary Figure 7). This third-order TG signal field with wavevector $\mathbf{k}_{TG} = \mathbf{k}_{pr}$ goes to the detector and it interferes with the preceding probe field, where the time delay of this TG signal field from the preceding probe pulse is, by definition, identical to the waiting time $|T_w|$. Therefore, regardless of the scanning delay time τ, the time delay between the probe pulse and the generated TG signal field is fixed at $|T_w|$. Since our detector measures the spectral interference, the interference between the two (probe and TG signal) would produce notable fringe pattern that is determined by the frequency of the electric field and time-delay $|T_w|$ along the y (ω_{pr}) axis in a given 2DES spectrum. Since detailed theoretical description for such three-pulse TG processes was presented previously,[7,8] we shall not provide any further theoretical description on the negative waiting time 2DES spectral interference patterns here.

Supplementary Methods

Sample preparation of LHC: The photosynthetic bacteria (Rhodobacter sphaeroides) in diluted sistrom medium A were cultured in a shaking incubator at 140 rpm at 31°C. The cultured photosynthetic bacteria were put into a 1 L bottle and centrifuged at 7000rpm at 4 °C for 15 min. The cell extract was harvested and stored at -80 °C until required. In order to separate intracytoplasmic membranes (ICMs), onto which a number of light-harvesting complexes (LHCs) are embedded, from the cell extract, 20g of cells were first dissolved in 20 mM HEPES and 5 mM EDTA (pH 7.5) buffer solution (per 20 ml). Cell breakage was performed by treatment with lysozyme (1mg/ml) for 30 min at 37 °C, then supplementation with a few crystals of DNase I prior to three passages through a French pressure. Unbroken cells were removed by centrifugation at 32,000×g at 4 °C for 20 min. The cell extract was layered onto a 15/40% (w/w) density-gradient sucrose and then, fractionated by centrifugation at 90,000×g at 4 °C for 12 h. The pigmented ICM band was isolated and used for 2DES measurement of LHC.

2DES measurement of LHC: The 2DES experimental condition is almost the same as that of AuNR. The perpendicular polarization configuration was employed to minimize a scattering signal from the sample. The FWHM of the cross-correlation of pump and probe pulses ranges from 75-80 fs to 90-100 fs, depending on the center frequency.

Supplementary References

1. Song, K.-H., Gu, M, Kim, M.-S., Kwon, H.-J., Rhee, H., Han, H. & Cho, M. Quantum Beats and Phase Shifts in Two-Dimensional Electronic Spectra of Zinc Naphthalocyanine Monomer and Aggregate. *J. Phys. Chem. Lett.* **6**, 4314-4318 (2015).
2. Gallagher Faeder, S. M. & Jonas, D. M. Two-dimensional electronic correlation and relaxation spectra: Theory and model calculations. *J. Phys. Chem. A* **41**, 10489-10505 (1999).
3. Rhee, H., Ha, J.-H., Jeon, S.-J. & Cho, M. Femtosecond spectral interferometry of optical activity: Theory. *J. Chem. Phys.* **129**, 094507 (2008).
4. Rhee, H., June, Y.-G., Lee, J.-S., Lee, K.-K., Ha, J.-H., Kim, Z. H., Jeon, S.-J. & Cho. M. Femtosecond characterization of vibrational optical activity of chiral molecules. *Nature* **458**, 310-313 (2009).
5. Rhee, H., Eom, I., Ahn, S.-H. & Cho, M. Coherent electric field characterization of molecular chirality in the time domain. *Chem. Soc. Rev.* **41**, 4457-4466 (2012).
6. Eom, I., Ahn, S.-H., Rhee, H. & Cho, M. Single-Shot Electronic Optical Activity Interferometry: Power and Phase Fluctuation-Free Measurement. *Phys. Rev. Lett.* **108**, 103901 (2012).
7. Cho, M. *Two-dimensional optical spectroscopy* (CRS Press, 2009).
8. Mukamel, S. *Principles of Nonlinear Optical Spectroscopy* (Oxford University Press, 1995).
