# Supplementary material for: Electron heating and thermal relaxation of gold nanorods revealed by two-dimensional electronic spectroscopy
Source: Nat Commun. 2018 Mar 1;9:891. doi: 10.1038/s41467-018-03002-8 (PMC5832861; doi:10.1038/s41467-018-03002-8)
Supplement: Supplementary file 3 — Description of Additional Supplementary Files [file 41467_2018_3002_MOESM3_ESM.pdf]

Description of Supplementary Files

File Name: Supplementary Movie 1
Description: Time-dependent two-dimensional electronic spectra. The waiting time varies from 0 to 500 fs. The pump (excitation) and probe (detection) wavelengths are 720 nm.

File Name: Supplementary Movie 2
Description: Time-dependent two-dimensional electronic spectra. The waiting time varies from 0 to 500 fs. The pump (excitation) and probe (detection) wavelengths are 740 nm.

File Name: Supplementary Movie 3
Description: Time-dependent two-dimensional electronic spectra. The waiting time varies from 0 to 500 fs. The pump (excitation) and probe (detection) wavelengths are 760 nm.

File Name: Supplementary Movie 4
Description: Time-dependent two-dimensional electronic spectra. The waiting time varies from 0 to 500 fs. The pump (excitation) and probe (detection) wavelengths are 790 nm.

File Name: Supplementary Movie 5
Description: Time-dependent two-dimensional electronic spectra. The waiting time varies from 0 to 500 fs. The pump (excitation) and probe (detection) wavelengths are 810 nm.
